# Risk of malignancy in patients with psoriasis according to treatment modalities in Korea: a nationwide cohort study

**DOI:** 10.1038/s41598-022-23518-w

**Published:** 2022-11-30

**Authors:** Ji Youn Hong, Juhee Ahn, Sungho Won, Sung Min Kim, Young Ah Cho, Chang Yong Kim, Jae Young Sung, Da-Ae Yu, Yang Won Lee, Yong Beom Choe

**Affiliations:** 1 Department of Dermatology, Konkuk University School of Medicine, 120-1 Neungdong-ro, Gwangjin-gu, Seoul, 143-729 Republic of Korea; 2 Department of Public Health Sciences, Seoul National University, Seoul, Republic of Korea; 3 Institue of Health and Environment, Seoul National University, Seoul, Republic of Korea; 4 RexSoft Inc., Seoul, Republic of Korea; 5 Research Institute of Medical Science, Konkuk University School of Medicine, Seoul, Republic of Korea

**Keywords:** Diseases, Risk factors

## Abstract

Intrinsic immunologic disparity of psoriasis itself, along with chronic inflammation and immunomodulatory anti-psoriatic treatments could be associated with increased risk of malignancy. We aimed to estimate the risk of malignancy in patients with psoriasis by treatment modality compared with that in individuals without psoriasis in Korea. We conducted a nationwide cohort study using the claims database of the National Health Insurance Service from January 2005 to December 2018. A total of 255,471 patients with psoriasis, and age- and sex-matched non-psoriasis participants (1:1 ratio) were enrolled. The adjusted hazard ratios (aHRs) [95% confidence intervals (CIs)] for malignancy without nonmelanoma skin cancer (NMSC) were 1.10 [1.08–1.12] in patients with psoriasis, 1.13 [1.00–1.27], 1.05 [0.97–1.13], and 1.24 [0.84–1.83] in phototherapy, non-biologic systemics, and biologics cohort, respectively. Among the non-biologic systemics cohort, patients treated with cyclosporin showed higher risk of malignancy without NMSC (aHR [95% CI], 1.20 [1.04–1.39]). The risk of malignancy without NMSC in patients with psoriasis was higher than that in individuals without psoriasis. Phototherapy and biologics were not associated with significant increase of risk; however, cyclosporin appeared to increase its risk. Dermatologists should be vigilant about this potential risk while managing patients with psoriasis.

## Introduction

Psoriasis is a chronic immunological skin disease mediated by T-helper (Th) 1 and Th17 cells^1^. Its prevalence in the United States and Europe is 1.3–3.0%, while 0.45% of the population in Korea is affected by psoriasis^2^. Conventionally, phototherapy and systemic drugs, such as acitretin, cyclosporin, and methotrexate have been widely used for the treatment of psoriasis. As the immunologic pathophysiology of psoriasis is being more clearly elucidated, biologics, such as tumor necrosis factor (TNF)-α inhibitors, interleukin (IL)-12/23 inhibitors, and IL-17 inhibitors, have recently emerged as main treatment options for psoriasis^3–5^.

Malignancy is the leading cause of death globally, with an estimated 10 million deaths attributed to malignancy in 2020^6^. In Korea, the burden of malignancy is increasing with an aging society^7^. Intrinsic immunologic disparity of psoriasis itself, along with chronic inflammation and immunomodulatory systemic treatments may contribute to increased risk of malignancy in patients with psoriasis^8^. Although several studies have alluded to the association of psoriasis with increased risk of malignancy due to systemic treatments^4,9,10^, no definite theory has been established yet.

Several studies have reported inconsistent results on the association between psoriasis and malignancy. A cohort study reported that patients with psoriasis had an elevated risk of malignancy compared with the general population. However, there was no significant difference in the occurrence of malignancy based on treatment modalities, including non-biologic systemic therapies, phototherapy, and biologics^10^. On the other hand, another population-based cohort study conducted in Korea demonstrated that the risk of malignancy, especially gastric cancer, increased in patients with psoriasis compared with that in the control. Reportedly, the risks of non-Hodgkin lymphoma and nonmelanoma skin cancer (NMSC) increased only in patients undergoing systemic treatment^11^. Another previous study found that biologics did not increase the incidence of overall malignancy, but that they might be associated with an increased risk of NMSC, especially squamous cell carcinoma^12^. These discursive results of previous studies may be attributed to different definitions of the disease, inconsistent classification of cohorts, various demographic characteristics, and meager sample sizes.

Recently, population-based research on the association between psoriasis and malignancy has been expanding worldwide; however, inadequate data are available on the effect of individual anti-psoriatic treatments, particularly few cohort studies have been conducted in Asia. Furthermore, no study has evaluated the cumulative dosage of each treatment before the occurrence of malignancy. In this nationwide cohort study, we aimed to estimate the risk of malignancy in patients with psoriasis according to each treatment modality compared with that in individuals without psoriasis in Korea. Furthermore, we evaluated the real-world cumulative average dosage of anti-psoriatic treatment to verify the effect of each treatment more quantitatively.

## Patients and methods

### Data source

The study population was selected from the National Health Insurance Service-National Health Information Database (NHIS-NHID, [Research No. NHIS-2019-1-559])^13^. In Korea, national health insurance is compulsory by law, and NHIS is a single-payer organization. NHIS provides comprehensive datasets including 99% of claims data from healthcare providers and healthcare information of both inpatients and outpatients, regarding demographics, diagnoses, and prescriptions.

### Study population

In this population-based cohort study, we selected prevalent patients with psoriasis aged ≥ 18 years from January 1, 2005 to December 31, 2018, from the NHIS-NHID. Psoriasis was diagnosed based on the following International Classification of Diseases 10th revision (ICD-10) codes; L40, M070, M071, M072, M073, and M090. Each patient was matched with one participant without psoriasis according to age and sex from the general population. The index date for patients with psoriasis was defined as the end date of anti-psoriatic treatments for 12 weeks or the enrollment date for psoriasis if the patient does not have a prescription for systemic treatment. We considered a 365-day washout period before the initiation of each anti-psoriatic treatment. The same index date was considered for their matched non-psoriasis participants. We excluded patients diagnosed with any malignancy or human immunodeficiency virus infection and those who underwent organ transplantation before the index date. Each patient was followed from the index date until the clinical outcome occurred, or either until the date of death or the end of the study.

### Definition of exposures

The study population comprised of patients with psoriasis and matched individuals without psoriasis. Patients with psoriasis were classified as exclusive treatment cohorts; systemics (phototherapy, non-biologic systemics, and biologics) and non-systemics cohorts. The brief stream of cohort classification is as follows: Patients who have ever received biologics at least once were included in the biologics cohort. Among the biologic-naive patients, patients who have ever been treated with acitretin, cyclosporin, or methotrexate were assigned to the non-biologic systemics cohort. Patients in the phototherapy cohort were identified as individuals who received phototherapy alone. Patients who had never received the above systemic treatments were classified into the non-systemics cohort. For subgroup analyses, the biologics cohort was categorized into TNF-α inhibitor (adalimumab, etanercept, and infliximab), IL-12/23 inhibitor (guselkumab and ustekinumab), and IL-17 inhibitor (ixekizumab and secukinumab) subcohorts. Similarly, the non-biologic systemics cohort was divided into three subcohorts: acitretin, cyclosporin, and methotrexate. Exposure to each treatment was defined as receiving the treatment for ≥ 12 weeks, and we excluded patients who received < 12 weeks of treatment.

### Clinical outcome

The main outcomes were defined as the overall new onset of malignant neoplasms (ICD-10 codes: C00–C97) and in-situ neoplasms (D00–D09), except NMSC (C44, D04). The details of organ-specific 23 malignancy types are shown in Supplementary Table S1^7^.

### Potential confounders

We adjusted for potential confounders to obtain unbiased estimates of the main exposure. We considered age, sex, body mass index (BMI), smoking status, family history of malignancy, alcohol use disorder, and Charlson Comorbidity Index as potential confounders. Using ICD-10 code, alcohol use disorder was indicated by F10, with admission days (≥ 1) or outpatient department days (≥ 1).

### Statistical analysis

The baseline characteristics of the study population are described for all covariates. Analysis of variance and Pearson *χ*^2^/Fisher exact test were performed for continuous and categorical variables, respectively. Difference in age at onset of clinical outcomes by treatment was compared using the Kaplan–Meier method and log-rank test. Multivariable proportional hazard ratios, after adjusting for potential confounders, were calculated using Cox proportional hazard regression. Proportional hazard assumption was confirmed using the trend of Schoenfeld residuals. Any treatment cohorts were excluded from the analysis if the number of outcomes was < 5, since there was no sufficient statistical power. We also categorized participants according to treatments, and subgroup analyses were conducted similarly. Model comparison was performed using loglikelihood ratio test (LRT) to identify the differences among non-psoriasis participants, systemics, and non-systemics cohorts. We estimated the mean of cumulative dosages of each anti-psoriatic treatment from the initiation date of each treatment until the occurrence of malignancy without NMSC. Statistical significance was set at a *P*-value of < 0.05, and we corrected it using false discovery rate (FDR) for multiple comparisons. All tests were performed on SAS Enterprise Guide (version 7.15; SAS Institute Inc., Cary, NC, USA), R software (version 4.0.3; R project, Vienna, Austria), and Rex (version 3.5.3; RexSoft, Seoul, Republic of Korea)^14^.

### Ethical approval

This study was approved by the Institutional Review Board in Konkuk University Medical Center (KUMC-2019-03-004-002) and carried out in accordance with all relevant guidelines and regulations. Written consent was not necessary since personal information was encoded and researchers could only access it through remote control.

## Results

### Demographics

We identified 255,471 patients diagnosed with psoriasis in 2005–2018 and 255,471 matched non-psoriasis participants. The mean (standard deviation [SD]) of their follow-up time was 6.6 (3.8) years. There were 11,091 and 244,380 patients with psoriasis in the systemics and non-systemics cohorts, respectively. In the systemics cohort, 2881 patients underwent phototherapy, and 7,678 and 532 patients underwent treatment with non-biologics and biologics, respectively. Each summary of follow-up period is shown in Supplementary Table S2. The baseline characteristics of our study population are described in Table 1. Each mean of all covariates, except family history of malignancy, significantly differed between psoriasis patients and non-psoriasis participants.

**Table 1:** Baseline characteristics of the study population.

Characteristics	Non-psoriasis (*n* = 255,471)	Patients with psoriasis				*P*-value
		Non-systemics (*n* = 244,380)	Phototherapy (*n* = 2,881)	Non-biologic systemics (*n* = 7,678)	Biologics (*n* = 532)	
**Age, years, Mean (SD)**	51.9 (13.8)	52.0 (13.9)	47.4 (12.8)	51.1 (12.2)	48.2 (10.9)	** < 0.001**
**Sex, n (%)**						** < 0.001**
Male	139,200 (54.5%)	132,065 (54.0%)	1642 (57.0%)	5104 (66.5%)	389 (73.1%)	
Female	116,271 (45.5%)	112,315 (46.0%)	1239 (43.0%)	2574 (33.5%)	143 (26.9%)	
**BMI, kg/m**^**2**^, **Mean (SD)**	23.7 (3.2)	23.9 (3.2)	23.5 (3.2)	24.1 (3.2)	24.3 (3.4)	** < 0.001**
**Smoking, n (%)**						** < 0.001**
Never	167,390 (65.5%)	157,645 (64.5%)	1722 (59.8%)	3703 (48.2%)	250 (47.0%)	
Ever	22,744 (8.9%)	22,245 (9.1%)	271 (9.4%)	723 (9.4%)	46 (8.6%)	
Current	65,337 (25.6%)	64,490 (26.4%)	888 (30.8%)	3252 (42.4%)	236 (44.4%)	
**Family history of malignancy, n (%)**						0.360
No	223,252 (87.4%)	213,648 (87.4%)	2,485 (86.3%)	6,701 (87.3%)	471 (88.5%)	
Yes	32,219 (12.6%)	30,732 (12.6%)	396 (13.7%)	977 (12.7%)	61 (11.5%)	
**History of alcohol use disorder, n (%)**	2693 (1.1%)	3032 (1.2%)	29 (1.0%)	109 (1.4%)	12 (2.3%)	** < 0.001**
**CCI, *n* (%)**						** < 0.001**
0	139,767 (54.7%)	112,290 (45.9%)	1524 (52.9%)	3353 (43.7%)	205 (38.5%)	
1	62,677 (24.5%)	65,310 (26.7%)	748 (26.0%)	2232 (29.1%)	153 (28.8%)	
2	28,063 (11.0%)	33,151 (13.6%)	300 (10.4%)	1071 (13.9%)	105 (19.7%)	
3	12,797 (5.0%)	16,211 (6.6%)	152 (5.3%)	524 (6.8%)	37 (7.0%)	
4	6351 (2.5%)	8674 (3.5%)	75 (2.6%)	263 (3.4%)	13 (2.4%)	
$\ge$ 5	5816 (2.3%)	8744 (3.6%)	82 (2.8%)	235 (3.1%)	19 (3.6%)	

Significant values are in bold.

BMI, body mass index; CCI, Charlson Comorbidity Index; SD, standard deviation.

### Psoriasis and risk of malignancy without NMSC

**Figure 1:**
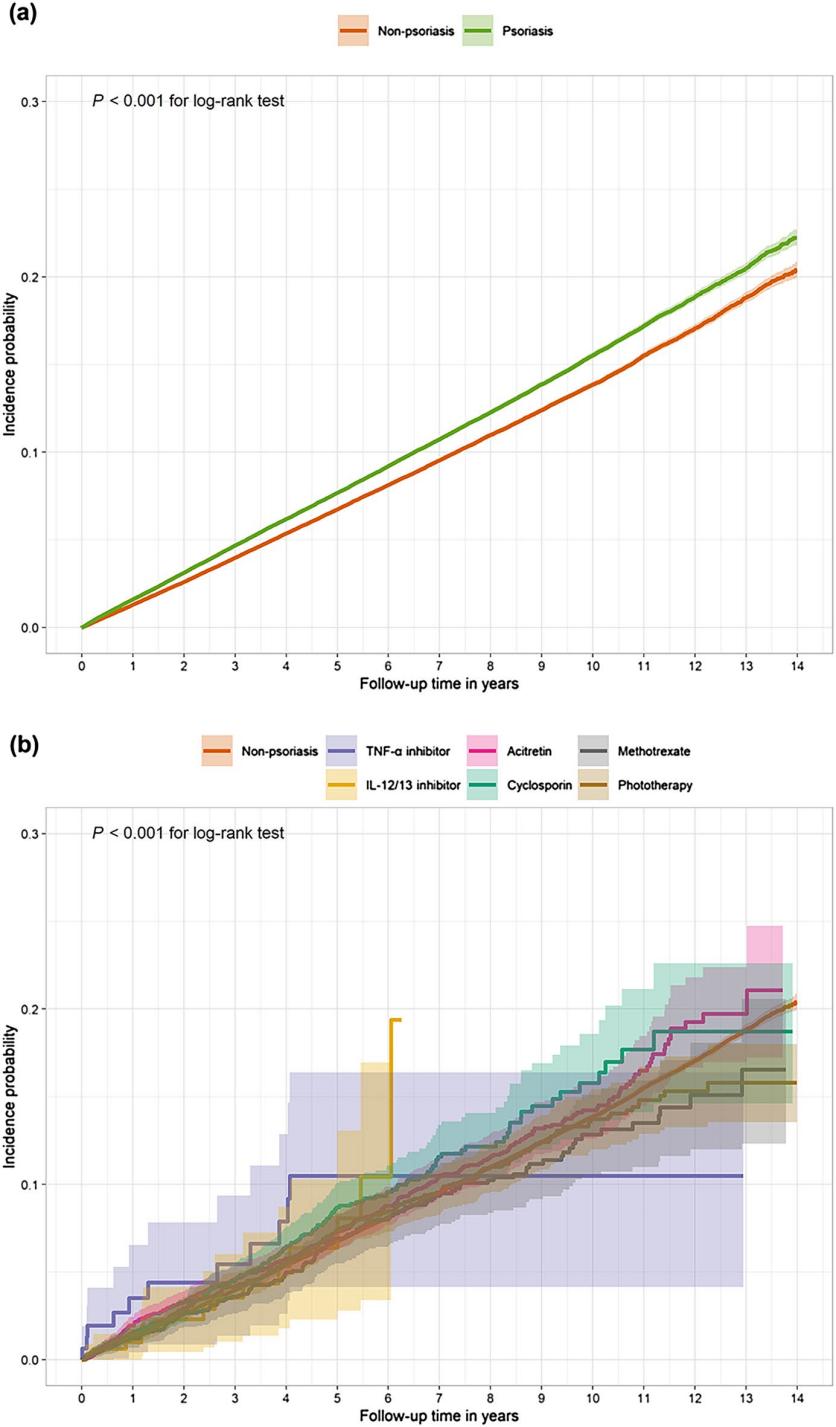
Incidence probabilities for psoriasis and non-psoriasis participants according to anti-psoriatic treatments. Comparison of Kaplan–Meier curve on malignancy without NMSC between (**a**) psoriasis and non-psoriasis participants and (**b**) psoriasis according to anti-psoriatic treatments and non-psoriasis participants. IL-12/23, interleukin-12/23; NMSC, nonmelanoma skin cancer; TNF-α, tumor necrosis factor-α.

Table 2 shows the risk estimates of malignancy without NMSC between psoriasis patients and non-psoriasis participants, and those for the 23 malignancy types are shown in Supplementary Table S3. The risk of malignancy without NMSC was significantly higher in patients with psoriasis than in non-psoriasis participants (Fig. 1a; *P* < 0.001 for log-rank test, Table 2; adjusted hazard ratio (aHR) [95% confidence interval (CI)], 1.10 [1.08–1.12]).

**Table 2:** Risk of malignancy without NMSC in patients with psoriasis according to anti-psoriasis treatments compared with in non-psoriasis participants and their goodness-of-fit statistics.

Risk of malignancy without NMSC			
Treatments	Event	aHR [95% CI]^†^	
Non-psoriasis (ref)	24,901	1.00	
Patients with psoriasis	27,878	1.10 [1.08–1.12]^‡^	
Non-systemics	26,937	1.10 [1.08–1.12]^‡^	
Systemics	941	1.07 [1.00–1.14]^‡^	
Phototherapy	261	1.13 [1.00–1.27]	
Non-biologic systemics	655	1.05 [0.97–1.13]	
Biologics	25	1.24 [0.84–1.83]	

Goodness-of-fit			
Model	df	${\chi }^{2}$	*P*-value
Non-psoriasis/patients with psoriasis			
Non-psoriasis/non-systemics/systemics	1	0.692	0.450

^†^Adjusted for age, sex, BMI, smoking status, family history of malignancy, Charlson Comorbidity Index, and alcohol use disorder (F10).

^‡^The null hypothesis was rejected at a significance level of 0.05.

aHR, adjusted hazard ratio; BMI, body mass index; CI, confidence interval; NMSC, nonmelanoma skin cancer; ref, reference.

**Figure 2:**
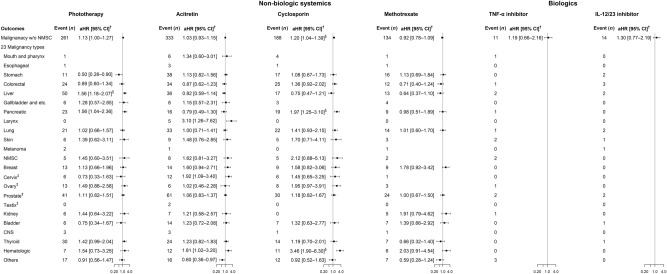
Risk of malignancy without NMSC in patients with psoriasis according to subcohorts of anti-psoriatic treatments compared with non-psoriasis participants. ^†^Adjusted for age, sex, BMI, smoking status, family history of malignancy, Charlson Comorbidity Index, and alcohol use disorder (F10). ^‡^Adjusted for age, BMI, smoking status, family history of malignancy, Charlson Comorbidity Index, and alcohol use disorder (F10). ^§^The null hypothesis was rejected at false discovery rate adjusted *P*-value of 0.05. aHR, adjusted hazard ratio; BMI, body mass index; CI, confidence interval; CNS, central nervous system; IL-12/23, interleukin-12/23; NMSC, nonmelanoma skin cancer; TNF-α, tumor necrosis factor-α; w/o, without.

The systemics and non-systemics cohort had significantly increased aHRs of malignancy without NMSC compared with non-psoriasis participants (Table 2; systemics: aHR [95% CI], 1.07 [1.00–1.14]; non-systemics: 1.10 [1.08–1.12﻿]). However, there was no significant difference between the systemics and non-systemics cohorts (*P* = 0.450 for LRT). Moreover, phototherapy (aHR [95% CI], 1.13 [1.00–1.27]), non-biologic systemics (1.05 [0.97–1.13]), and biologics (1.24 [0.84–1.83]) cohorts were not associated with a significantly increased risk of malignancy without NMSC compared with non-psoriasis participants.

We compared the risk of malignancy without NMSC between subcohorts based on anti-psoriatic treatments, regarding the 23 specific malignancy types shown in Figs. 1b and 2. Risk estimates on malignancy without NMSC in phototherapy were not significant, as in the case of skin cancer (aHR [95% CI], 1.39 [0.62–3.11]) and NMSC (1.45 [0.60–3.51]). For acitretin and methotrexate users, the aHR of malignancy without NMSC was 1.03 (95% CI, 0.93–1.15) and 0.92 (0.78–1.09), respectively. Even in all 23 malignancy subtypes, non-significant results were observed at FDR-adjusted *P*-values. For cyclosporin, the risk of malignancy without NMSC was higher (aHR [95% CI], 1.20 [1.04–1.39]) than that in non-psoriasis participants, including hematologic (3.46 [1.90–6.30]) and pancreatic (1.97 [1.25–3.10]) cancers. In the biologics cohort, the risk of malignancy without NMSC was not significantly high, and this trend was similar in the TNF-α inhibitor and IL-12/23 inhibitor cohorts (aHR [95% CI], 1.19 [0.66–2.16] and 1.30 [0.77–2.19], respectively).

### Cumulative dosage of each anti-psoriatic treatment

Table 3 summarizes the cumulative dosages of anti-psoriatic treatments among patients who experienced malignancy without NMSC. Mean (SD) cumulative dosages were 44.1 (46.7) for phototherapy, 4970.7 (5649.2) mg for acitretin, 35054.7 (50603.5) mg for cyclosporin, 471.5 (709.1) mg for methotrexate, 1154.3 (861.8) mg for adalimumab, 5765.0 (4688.1) mg for etanercept, 5000.0 (5798.3) mg for infliximab, and 507.9 (329.0) mg for ustekinumab.

**Table 3:** Cumulative dosage of each anti-psoriatic treatment between the initiation date of each treatment and malignancy without NMSC.

Treatment	Dosage		
	Event (*n*)	Mean	SD
**Phototherapy**	261	44.1	46.7
**Non-biologic systemics**			
Acitretin (mg)	333	4970.7	5649.2
Cyclosporin (mg)	188	35054.7	50603.5
Methotrexate (mg)	134	471.5	709.1
**Biologics**			
Adalimumab (mg)	7	1154.3	861.8
Etanercept (mg)	2	5765.0	4688.1
Infliximab (mg)	2	5000.0	5798.3
Ustekinumab (mg)	14	507.9	329.0
Guselkumab (mg)	0	–	–
Secukinumab (mg)	0	–	–
Ixekizumab (mg)	0	–	–

NMSC, nonmelanoma skin cancer; SD, standard deviation.

## Discussion

In this study, patients with psoriasis showed higher risk of malignancy without NMSC than non-psoriasis participants. However, we found no significant difference in the risk of malignancy between patients with psoriasis undergoing systemic therapy and those who were not. Inflammation is known to have an ambivalent effect on the development of malignancy^15^. Chronic inflammation can cause DNA damage and proinflammatory cytokines, including TNF, IL-1, and IL-6, and can augment tumor growth and metastasis. Further, immunosurveillance by activation of inflammatory cells inhibits tumorigenesis. According to our results, pro-tumorigenic effects seem to overwhelm the anti-tumor responses in chronic inflammatory status of psoriasis.

Phototherapy using ultraviolet (UV) wavelengths is a well-established treatment for psoriasis. It decreases dendritic cell activity and inhibits effector T-cell activation, which is effective in managing psoriasis^16,17^. However, phototherapy raises concerns of an increased risk of skin cancer. Several studies revealed that psoralen and ultraviolet A (PUVA) could increase the risk of skin cancer in a dose-dependent manner, without affecting risk of internal organ malignancies^18–20^. Conversely, broad- or narrow-band UVB was known to have no effect on malignancies, including skin cancer^21,22^. This study reported that phototherapy did not significantly increase skin cancer, including NMSC. It may result from the frequent use of broad- or narrow-band UVB than PUVA for psoriasis treatment in the recent two decades. Unfortunately, we could not distinguish between the use of broad- or narrow-band UVB from PUVA due to the limitations in claims data. However, it was supported by a previous Korean study by Song et al.^23^, which revealed that among 1260 patients with psoriasis, 45.6% received broad- or narrow-band UVB phototherapy, whereas only 3.3% received PUVA.

Cyclosporin is a calcineurin inhibitor approved for psoriasis treatment. It binds the intracytoplasmic receptor, cyclophilin, and forms the cyclosporin–cyclophilin complex. This complex selectively disrupts T-cell functions, which has potent immunomodulatory effect^24^. The long-term use of cyclosporin can cause longstanding immunosuppression associated with an increased risk of malignancies including NMSC and lymphoproliferative cancer^25^. A rheumatology study reported an increased risk of lymphoproliferative malignancies in patients with severe rheumatoid arthritis treated with cyclosporin^26^. Moreover, especially in studies on organ transplantation, cyclosporin-associated skin cancer has been frequently documented^27,28^. It may result from T-cell dysfunction and upregulation of transforming growth factor-β production induced by cyclosporin, which increases the invasive activity of the cells^29^. Based on these observations, cyclosporin has not been recommended for patients with a high risk of malignancy^4^. However, a 5-year cohort study by Paul et al.^30^ reported no increased risk of malignancy in patients treated with cyclosporin, except NMSC. Even in that study, all patients who developed NMSC previously received PUVA before cyclosporin treatment. In the present study, cyclosporin had an increased risk of malignancy without NMSC, unlike other systemic treatments. It was mainly implicated with hematologic cancer and pancreatic cancer, and not skin cancer, including NMSC.

Methotrexate is also commonly used for psoriasis and psoriatic arthritis. It has immunosuppressive and anti-inflammatory effects via the inhibition of dihydrofolate reductase, which may be associated with a higher risk of malignancy. One meta-analysis that evaluated the safety and efficacy of methotrexate in patients with psoriasis reported that the incidence of malignancy was 1.2%^31^. Among the results from several rheumatology studies, methotrexate did not significantly increase the incidence of malignancy in patients with rheumatoid arthritis, including lymphoproliferative cancer and skin cancer^32–34^. In the present study, methotrexate showed a more reliable safety profile for malignancy. There were no significant differences between patients treated with methotrexate and non-psoriasis participants in the development of malignancy, including all organ-specific malignancies, even in hematologic cancer, which is consistent with the results of the previous rheumatologic studies.

Acitretin is a synthetic retinoid with reliable pharmacokinetic features. It recovers epidermal proliferation and is remarkably effective in treating psoriasis. There are several reports on its prophylactic use for preventing skin cancer in post-transplantation patients^35,36^. Moreover, in combination therapy of acitretin and phototherapy for psoriasis treatment, acitretin is known to curtail the risk of skin cancer, which can be increased by phototherapy^37^. Although studies are sparse for evaluating the effect of acitretin on internal organ malignancy, acitretin may not increase the risk of malignancy because it lacks immunosuppressive properties. Our study failed to observe the protective effect of acitretin against skin cancer including NMSC, but acitretin did not significantly increase the risk of skin cancer in psoriasis. In addition, all malignancy subtypes did not increase in the acitretin cohort, compared with that in non-psoriasis participants.

The recent development in biologics blocking TNF-α, IL-12/23, and IL-17, which are key cytokines in psoriasis, contributes to promising outcomes for the treatment of psoriasis. TNF-α plays a central role in the inhibition of tumor growth^38^, and IL-12, IL-23, and IL-17 also have a protective effect against tumor progression^39^. Thus, there are theoretical risks of malignancy in patients treated with biologics inhibiting such proinflammatory cytokines. However, the long-term safety of biologics has not been established compared with other anti-psoriatic agents, and several studies have shown inconsistent results on the incidence of malignancy in patients with psoriasis^9,40,41^. In the present study, we did not find an increased risk of malignancy without NMSC in patients treated with biologics, nor in the TNF-α inhibitor and IL-12/23 inhibitor cohorts.

Our study has several limitations. This nationwide cohort study could not include clinical information regarding disease severity such as the Psoriasis Area and Severity Index score, and laboratory results. The misclassification of diagnosis may have occurred in the study based on the claims data. A previous study reported 70% concurrence between the diagnosis code from the NHIS database and clinical diagnosis^42^.

Patients treated with high degree medications such as biologics may be exposed to previous medications with high probability. Measurement error could have occurred due to previous medication exposure, which could have made groups seem more homogenous than actual. Moreover, since biologics have been rousingly prescribed recently and are mainly used for moderate-to-severe patients, few patients are exposed to biologics and their follow-up period is relatively short. Therefore, there is a potential fallacy that the risk of malignancy in the biologics cohort could be statistically misestimated. Since the psoriasis subgroups, including biologics cohort, have less samples compared to the total psoriasis group, statistical power to detect significant difference may become lower. In addition, since each organ-specific malignancy may have distinctive risk factors, individualized adjustments are required to analyze the risk of organ-specific malignancy.

However, our study has several strengths. This study is a nationwide cohort study that could mirror the characteristics of the entire population and includes a long-term follow-up period. Moreover, we included the health medical examination data such as BMI, smoking status, and alcohol consumption, and adjusted for the confounding factors. Our study has a great clinical significance in that we conducted comprehensive analyses on the risk according to each anti-psoriatic treatment individually. To minimize the impact of single or short-term exposure to each treatment, we only enrolled patients who used each treatment for ≥ 12 weeks. Furthermore, we can clarify the effect of each treatment on malignancy by rendering the average cumulative dosage.

In conclusion, this study revealed that patients with psoriasis might be at a higher risk of malignancy without NMSC than non-psoriasis participants. Phototherapy and biologics did not appear to increase the risk of malignancy without NMSC in patient with psoriasis. Unlike other anti-psoriatic systemic treatments, cyclosporin was associated with an increase in the risk. Dermatologists should deliberate these potential risks while managing patients with psoriasis.

## Supplementary Information

Supplementary Information.

## Data Availability

Data are available from the National Health Insurance-National Health Information Database (NHIS-NHID), Republic of Korea. Data may not be made publicly available without request.
