# Supplementary material for: Risk of malignancy in patients with psoriasis according to treatment modalities in Korea: a nationwide cohort study
Source: Sci Rep. 2022 Nov 30;12:20690. doi: 10.1038/s41598-022-23518-w (PMC9712678; doi:10.1038/s41598-022-23518-w)
Supplement: Supplementary file 1 — Supplementary Information. [file 41598_2022_23518_MOESM1_ESM.docx]

Supplementary Table S1. Definition of 23 malignancy types according to ICD-10 codes.

| **Codes** | **Definition** |
| --- | --- |
| C00–C14, D000 | Lip, oral cavity and pharynx |
| C15, D001 | Esophagus |
| C16, D002 | Stomach |
| C18-C20, D010–D012 | Colorectal |
| C22, D0150, D0152 | Liver |
| C23, C24, D0151, D0153 | Gallbladder and etc. |
| C25, D017 | Pancreatic |
| C32, D020 | Larynx |
| C33, C34, D021, D022 | Lung |
| C43, D03 | Melanoma |
| C44, D04 | Nonmelanoma skin cancer |
| C50, D05 | Breast |
| C53, C54, D06 | Cervix |
| C56 | Ovary |
| C61, D075 | Prostate |
| C62 | Testis |
| C64 | Kidney |
| C67, D090 | Bladder |
| C70–C72 | Central nervous system |
| C73, D0930 | Thyroid |
| C81–C83, C840, C841, C844–C847, C849, C85, C86, C90–C96 | Hematologic |

Abbreviation: ICD-10, International classification of diseases, 10^th^ revision.

Supplementary Table S2. Summary of follow-up duration.

|  | **Follow-up duration (years)** | | | |
| --- | --- | --- | --- | --- |
| **Treatment** | ***n*** | **Mean (SD)** | **Median** | **IQR** |
| ***Non-psoriasis*** | 255,471 | 6.6 (3.8) | 6.5 | 6.3 |
| ***Psoriasis*** | 255,471 | 6.5 (3.8) | 6.5 | 6.3 |
| *Non-systemics* | 244,380 | 6.6(3.8) | 6.5 | 6.3 |
| *Phototherapy* | 2,881 | 6.3 (3.8) | 6.2 | 6.5 |
| *Non-biologic systemics* | 7,678 | 5.5 (3.7) | 5.1 | 6.1 |
| Acitretin | 3,297 | 6.3 (3.7) | 6.4 | 6.1 |
| Cyclosporin | 2,655 | 4.2 (3.1) | 3.7 | 4.7 |
| Methotrexate | 1,726 | 5.8 (3.9) | 5.4 | 6.6 |
| *Biologics* | 532 | 2.9 (2.5) | 2.2 | 3.7 |
| TNF-α inhibitor | 154 | 4.1 (3.3) | 3.5 | 6.3 |
| IL-12/23 inhibitor | 344 | 2.5 (1.9) | 2.2 | 3.3 |
| IL-17 inhibitor | 34 | 0.5 (0.3) | 0.4 | 0.5 |

Abbreviation: IL-12/23, interleukin-12/23; IL-17, interleukin-17; **IQR, interquantial range; SD, stand**ard deviation; TNF-α, tumor necrosis factor-α.

Supplementary Table S3. Risk of 23 malignancy types in patients with psoriasis compared with non-psoriasis subjects.

|  | **Patients with psoriasis** | |
| --- | --- | --- |
| **23 malignancy types** | **Event (*n*)** | **aHR [95% CI]**^†^ |
| Mouth and pharynx | 490 | 1.33 [1.16–1.53]^§^ |
| Esophageal | 194 | 1.22 [0.99–1.50] |
| Stomach | 2,463 | 0.98 [0.92–1.03] |
| Colorectal | 3,201 | 1.05 [1.00–1.10] |
| Liver | 3,539 | 1.08^§^ [1.02–1.13] |
| Gallbladder and etc. | 622 | 1.06 [0.95–1.19] |
| Pancreatic | 1,863 | 1.14^§^ [1.06–1.22] |
| Larynx | 120 | 1.15 [0.89–1.51] |
| Lung | 2,790 | 1.11 [1.05–1.18]^§^ |
| Skin | 836 | 1.48 [1.33–1.65]^§^ |
| Melanoma | 130 | 1.23 [0.95–1.59] |
| NMSC | 710 | 1.53 [1.36–1.73]^§^ |
| Breast | 1,072 | 0.97 [0.90–1.06] |
| Cervix^‡^ | 751 | 1.04 [0.94–1.15] |
| Ovary^‡^ | 758 | 1.04 [0.94–1.15] |
| Prostate^‡^ | 5,039 | 1.19 [1.14–1.24]^§^ |
| Testis^‡^ | 57 | 1.04 [0.71–1.52] |
| Kidney | 506 | 1.14 [1.00–1.30] |
| Bladder | 998 | 1.09 [0.99–1.19] |
| CNS | 253 | 0.97 [0.82–1.16] |
| Thyroid | 2,228 | 1.13 [1.06–1.20]^§^ |
| Hematologic | 588 | 1.11 [0.98–1.25] |
| Others | 2,205 | 1.03 [0.97–1.09] |

^†^Adjusted for age, sex, BMI, smoking status, family history of malignancy, Charlson comorbidity index, and alcohol use disorder (F10).

^‡^Adjusted for age, BMI, smoking status, family history of malignancy, Charlson comorbidity index, and alcohol use disorder (F10).

^§^The null hypothesis was rejected at false discovery rate adjusted *P*-value of 0.05.

Abbreviation: aHR, adjusted hazard ratio; BMI, body mass index; CI, confidence interval; CNS, central nervous system; **NMSC, nonmelanoma skin cancer.**
